# Contrast‐enhanced spectral mammography: A potential exclusion diagnosis modality in dense breast patients

**DOI:** 10.1002/cam4.2877

**Published:** 2020-02-19

**Authors:** Yun Qin, Ying Liu, Xueqin Zhang, Shuang Zhao, Huanhuan Zhong, Juan Huang, Jianqun Yu

**Affiliations:** ^1^ Department of Radiology Sichuan University West China Hospital Chengdu China

**Keywords:** breast cancer, contrast‐enhanced spectral mammography, diagnostic efficiency, dynamic contrast‐enhanced MRI, pathology

## Abstract

**Background:**

China has an increasing burden of breast cancer. However, with a large population of dense breast patients, the diagnostic efficiency of conventional digital mammography is attenuated.

**Methods:**

From July 2017 to October 2018, we retrospectively reviewed 397 dense breast patients who underwent contrast‐enhanced spectral mammography (CESM) in West China Hospital. Among them, 53 patients who had both CESM and dynamic contrast enhanced magnetic resonance imaging (DCE‐MRI) results and 114 patients who had pathological diagnoses were finally enrolled. All images were reviewed by two independent radiologists according to the 2013 Breast Imaging Reporting and Data System (BI‐RADS) with all disagreements handed to an associate professor for final decisions. Correlation analyses between CESM and DCE‐MRI were conducted. The diagnostic performance of CESM were investigated.

**Results:**

The kappa value of the BI‐RADS scores between CESM and DCE‐MRI was 0.607 (*P* < .001), indicating high correspondence between CESM and DCE‐MRI. As for lesion size measurement, moderate correlation (Kendall's tau coefficient: 0.556, *P* < .001) was detected between CESM and DCE‐MRI. Using pathological diagnoses as the reference standard, the sensitivity, specificity, and area under the curve (AUC) of CESM were 82.4%, 96.4%, and 0.894, respectively.

**Conclusion:**

CESM demonstrated excellent overall diagnostic accuracy and a moderate correlation in lesion size estimation against DCE‐MRI in dense breast patients, supporting it to be an alternative to DCE‐MRI in breast cancer detection and diagnosis, especially for exclusion diagnosis.

## Graphical abstract

China has an increasing burden of breast cancer, but with a population of dense breast patients, the diagnostic efficiency of conventional digital mammography was attenuated. Our study suggested that CESM exhibited an excellent diagnostic efficiency and a moderate correlation in lesion size estimation compared to DCE‐MRI and pathology, supporting it to be an alternative to DCE‐MRI in breast cancer detection and diagnosis, especially for exclusion diagnosis in dense breast patients.

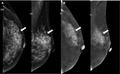

## 1 INTRODUCTION

Breast cancer (BC) is the most common cancer and the first leading cause of cancer‐related death among women worldwide.^1^ With an increasing burden of BC in China, there were 279 000 newly diagnosed patients with an age‐standard mortality rate of 6.35/100 000 in 2014, and the 5‐year survival rate (73.1%) was lower than that in Western countries, especially for those in rural areas (55.9%).^2^, ^3^ Early detection, diagnosis and treatment is crucial for improving the survival and life quality of BC patients.

According to the NCCN guideline, diagnostic bilateral mammography is recommended for BC patients of all stages (Category 2A). But in China with a large population of dense breast patients, the diagnostic efficiency of conventional digital mammography is attenuated. Dynamic contrast‐enhanced MRI (DCE‐MRI), because of its advantages in morphology and hemodynamics assessment, is recommended for evaluating the extent of BC, detecting the presence of multifocal or multicentric BC in the ipsilateral breast and screening of the contralateral BC at initial diagnosis (category 2B).^4^, ^5^ However, clinical application of DCE‐MRI could be limited because it is expensive both in time and cost, and requires specific conditions (eg, dedicated breast coils, the optimal timing sequences and breast imaging radiologists) for proper image acquisitions and interpretations.

First introduced by Lewin et al in 2001,^6^ contrast‐enhanced spectral mammography (CESM) enables effective combination of intravenous contrast enhancement and mammography. Using specific digital subtraction technique, CESM is able to erase the normal mammary gland and highlight the hypervascular lesions with better depiction of their frames and vasculatures. Preliminary studies have confirmed the superior sensitivity and specificity of CESM than conventional digital mammography.^7^, ^8^, ^9^ And several studies indicated the comparability of CESM to DCE‐MRI for breast cancer detection and diagnosis.^10^, ^11^ In addition, CESM is cheaper, less time‐consuming and more suitable for patients with metallic implant than DCE‐MRI. However, due to its recent introduction in clinical practice, especially in China, evidence regarding the role of CESM in BC detection and diagnosis remained limited.

The aim of our study was to evaluate CESM’s sensitivity and specificity in BC detection and diagnosis compared to DCE‐MRI and pathology. Meanwhile, using DCE‐MRI results as standard, evaluate the accuracy of CESM in lesion size estimation.

## 2 MATERIALS AND METHODS

### 2.1 Patients

This retrospective study was approved by the ethic committee of West China Hospital, and written informed consent was obtained from all patients. Form July 2017 to July 2018, dense breast patients who underwent CESM in West China Hospital were reviewed, and those who also underwent DCE‐MRI or had postoperative pathological results were enrolled. Dense breast patients were defined as patients classified as heterogeneously dense or extremely dense according to the 2013 Breast Imaging Reporting and Data System (BI‐RADS) by the American College of Radiology. In West China Hospital, CESM was carried out only when definite diagnoses couldn't be made by other examinations including ultrasound, mammography, or MRI.

### 2.2 Image acquisition and evaluation protocol

#### 2.2.1 CESM

CESM was performed using the GE healthcare equipment (SenoBright^®^) designed to collect the dual‐energy images. First, all patients received intravenous injection of iodine contrast media at a dose of 1.5 mL/kg. Two minutes after the injection, the standard bilateral breast images were obtained by the sequence of ipsilateral craniocaudal (CC) projection, contralateral CC projection, ipsilateral mediolateral (MLO) projection, and contralateral MLO projection. For each compression, both the low‐energy and high‐energy images were acquired with only 300‐ms delay. The final step was the CESM recombination algorithm which helped process the low‐energy and high‐energy images into iodine‐specific images.

#### 2.2.2 Dynamic contrast enhanced magnetic resonance imaging

All breast MR examinations were performed on a 3.0T MR scanner (DISCOVERY MR750w, GE Healthcare) with a dedicated phased‐array breast coil. Patients were in prone position without breast compression. Precontrast sequences included axial T1‐weighted imaging, axial and sagittal T2‐weighted imaging with fat suppression and diffusion‐weighted imaging. A precontrast axial high‐resolution T1‐weighted imaging with fat suppression was performed as mask, then following a 10‐second delay after intravenous injection of 0.2 mL/kg of gadolinium contrast media with dynamic contrast enhanced scanning performed 1‐2 times per minute. Image postprocessing was performed by software (Functool 2.0, GE Healthcare). Time‐intensity curve and MIP reconstruction imaging were achieved.

#### 2.2.3 CESM and DCE‐MRI evaluation

All MR images were evaluated according to the 2013 BI‐RADS. As for CESM imaging, we used a classification based on the 2013 BI‐RADS (scale 1‐5), in which the low‐energy images were evaluated just as the standard mammograms, while lesions in the iodine‐specific images were classified as enhancing (further classified as mass and nonmass) or nonenhancing. All imaging evaluations were performed by two independent radiologists who were blinded to any clinical and pathological data. All patient images were provided to the reviewers in random sequences, and both reviewers were asked to gap for at least one month between evaluating the CESM and DCE‐MRI images. All disagreements were handed to an associate professor who specialized in breast imaging for over 10 years for final judgment.

### 2.3 Statistical analysis

Kappa coefficient was used to assess the correlation of BI‐RADS scores between CESM and DCE‐MRI, and differences with regard to lesion diameter measurement between the two imaging modalities were evaluated using Kendall coefficient. To assess the diagnostic efficacy of CESM, per lesion sensitivity, specificity, and area under the receiver operating characteristic curve (AUC) were computed with postoperative pathological examination as the reference standard. CESM lesions scored less than 4C and MRI lesions scored less than 4 were considered as benign while others as malignant. Statistical analyses were performed using SPSS software (version 21.0, IBM) and *P* < .05 was considered to be statistically significant.

## 3 RESULTS

We retrospectively reviewed 397 dense breast patients who underwent CESM in West China Hospital from July 2017 to October 2018, and enrolled 53 patients who had both CESM and DCE‐MRI results, 114 patients who had pathological diagnoses and 36 patients with no pathological results but complete follow‐up records.

### 3.1 Comparisons between CESM and DCE‐MRI

There were 53 patients who had the results of CESM and DCE‐MRI simultaneously. Among these patients, only 17 (32.1%) had pathological results, whereas 24 of the remaining 36 patients were scored less than 4C. The comparisons between CESM and DCE‐MRI are shown in Table 1.

**Table 1:** The clinical and imaging features in the 53 cases

	CESM	DCE‐MRI
Age (y, mean [range])	44.5 (15‐74)	
Postoperative	6	
Results		
Negative^a^	23	22
Positive^b^	36	37
Laterality^b^		
Right	16	19
Left	20	18
Location^b^		
Upper Outer Quadrant	15	12
Upper Inner Quadrant	2	3
Lower Outer Quadrant	1	2
Lower Inner Quadrant	2	1
Central	15	18
Axilla	1	1
BI‐RADS^a^		
1	4	4
2	2	1
3	10	13
4	29	27
5	0	0
Other	8	8
Diameter (cm, mean [range])	2.1 (0.7‐3.4)	1.7 (0.7‐3.1)

Abbreviations: CESM, contrast‐enhanced spectral mammography; DCE‐MRI, dynamic contrast magnetic resonance imaging.

a Negative, BI‐RADS showed the number of patients.

b Positive, Laterality, Location showed the number of lesions.

**Figure 1:**
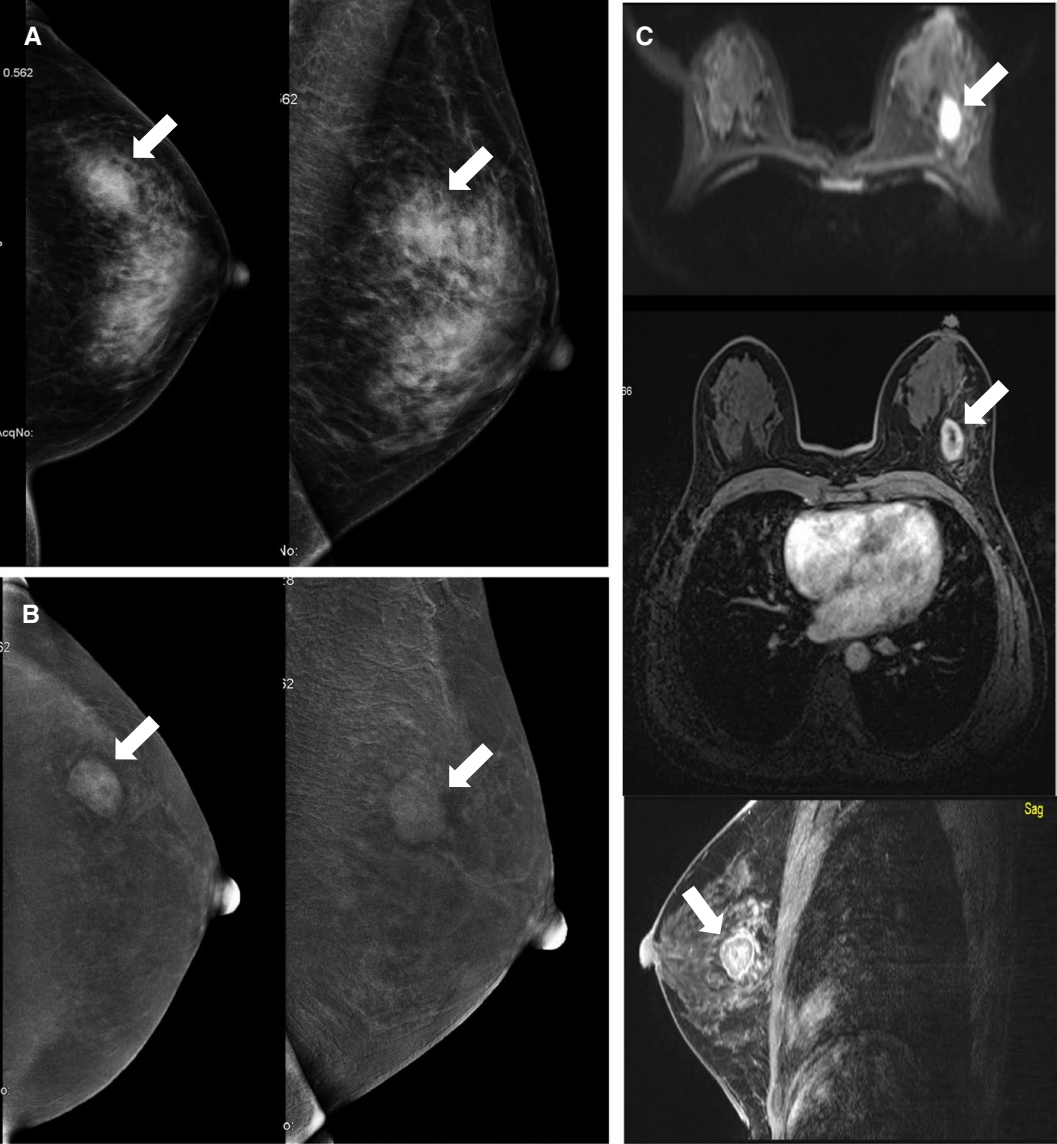
Breast images of a 40‐y‐old patient with a palpable mass in the left breast for 1 mo. A, conventional digital mammography showed focal asymmetry (white arrow) in the upper outer quadrant of the left breast (BI‐RADS 4B). B, Contrast‐enhanced spectral mammography images exhibited moderate‐enhanced mass (white arrow) with surrounding edema zone in the same area (BI‐RADS 4C). C, An early inhomogeneous enhanced and diffusion‐restricted mass (white arrow) on dynamic contrast magnetic resonance imaging. The pathology of biopsy reported invasive ductal carcinoma

**Figure 2:**
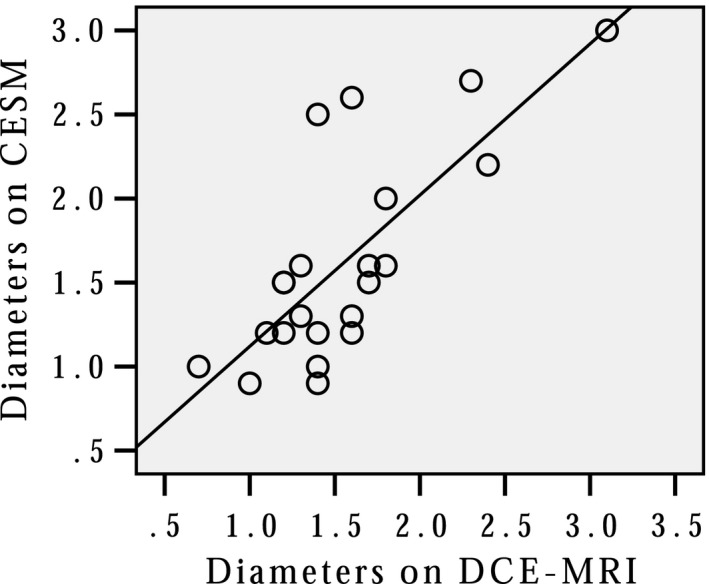
The Lesion Size Correlation between contrast‐enhanced spectral mammography and dynamic contrast magnetic resonance imaging

Of the 53 patients, 25 lesions in 23 (43.4%) patients were identified in consensus according to CESM and DCE‐MRI (Figure 1), 16 (30.2%) patients got negative results by consensus, and the remaining 14 (26.4%) patients encountered disagreement. The kappa value of the BI‐RADS scores between CESM and DCE‐MRI was 0.607 (*P* < .001), indicating high correspondence of the two imaging modalities. As for lesion size measurement, CESM and DCE‐MRI had a moderate correlation with a Kendall's tau coefficient of 0.556 (*P* < .001) (Figure 2).

### 3.2 Diagnostic accuracy of CESM

The main characteristics of the 114 patients was presented in Table 2.

**Table 2:** The main features of the 114 patients with pathological results

Age (y)	48, 18‐77
Laterality^a^	
Right	50
Left	94
Location^a^	
Upper Outer Quadrant	77
Upper Inner Quadrant	16
Lower Outer Quadrant	7
Lower Inner Quadrant	8
Central	32
Other	4
Specimens^a^	
Biopsy	59
Surgery	85
Pathological diagnoses^a^	
Ductal carcinoma	31
Secretory carcinoma	2
Paget's disease	1
Adenosis/Fibroadenoma	110

a Laterality, Location, Specimens and Pathological diagnoses all showed the number of lesions.

**Figure 3:**
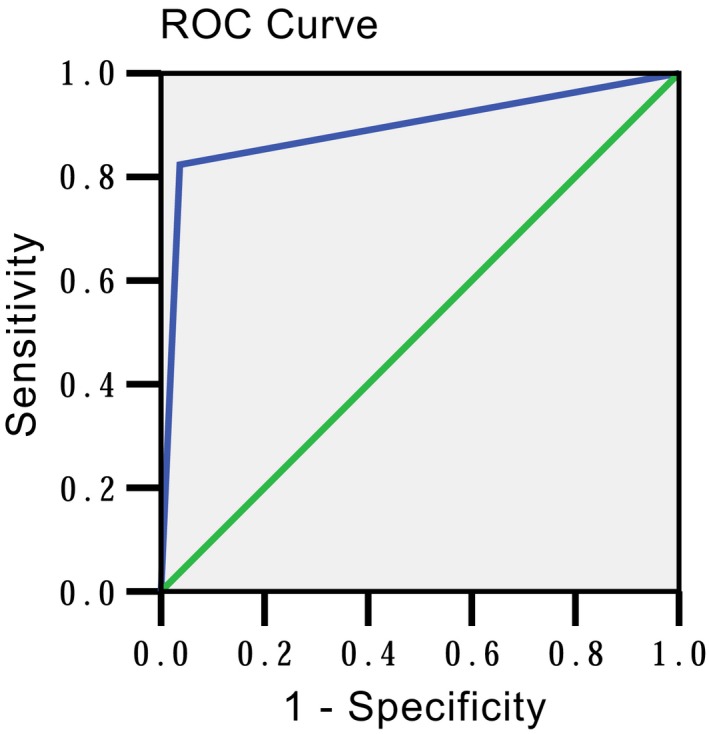
The ROC curve for contrast‐enhanced spectral mammography based on BI‐RADS scores

**Figure 4:**
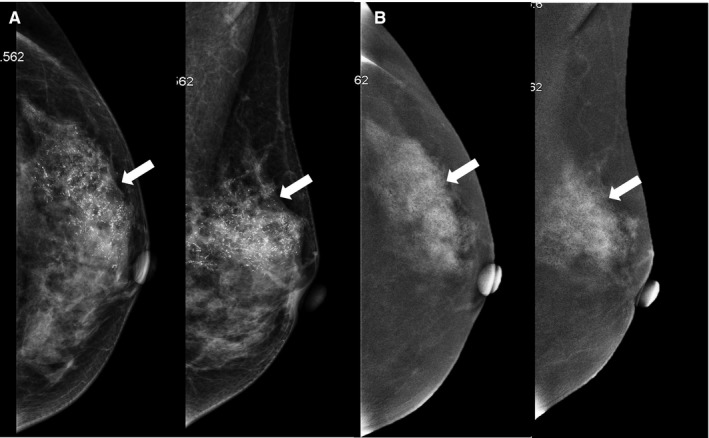
Breast images of a 47‐y‐old patient with a growing palpable mass in the left breast for 9 mo and erosion of the left nipple for 20 d. A, Conventional digital mammography showed amorphous calcification (white arrow) with architectural distortion in the upper outer quadrant of the left breast (BI‐RADS 4C). B, Contrast‐enhanced spectral mammography images exhibited severe patchy enhancement (white arrow) in the same area (BI‐RADS 5). The patient finally underwent left mastectomy and lymph node dissection of left axilla and infraclavicular region. The postoperative pathology reported invasive ductal carcinoma with axillary lymph node metastasis and Paget’s disease of the nipple

Of the 114 patients with 144 pathological proven lesions, 36 (31.6%) patients showed no visible lump or abnormal enhancement and got hyperplasia diagnoses with BI‐RADS scores ≤3. Their pathological results were adenosis with or without fibroadenoma. The sensitivity, specificity and AUC of CESM for benign and malignant lesion discrimination were 82.4%, 96.4%, and 0.894 (95%CI [0.815‐0.972], *P* < .001), respectively (Table 3 and Figures 3 and 4).

**Table 3:** The diagnostic accuracy of contrast‐enhanced spectral mammography

		Pathology	
		Malignant	Benign
CESM	Malignant	28	4
	Benign	6	106

	Value %	95% Confidence interval
Sensitivity	82.4	64.8‐92.6
Specificity	96.4	90.4‐98.8
True positive	87.5	70.1‐95.9
False positive	12.5	4.1‐29.9
True negative	94.6	88.2‐97.8
False negative	5.4	2.2‐11.8

## 4 DISCUSSION

DCE‐MRI, because of its advantages in morphology and hemodynamics assessment, is generally accepted as the most sensitive examination for breast disease in state‐of‐the‐art clinical practices. In the study published by Jochelson et al, both CESM and DCE‐MRI detected 96% of the index tumors, with 2 and 13 false‐positive findings, respectively, indicating CESM and DCE‐MRI had comparable detection rate, while CESM had higher specificity.^12^ Using histopathologically measured tumor size as the reference, Lobbes’ et al confirmed that the Pearson's correlation coefficients of CESM and DCE‐MRI were both greater than 0.9 (*P* < .0001), suggesting excellent tumor size measurement ability of both CESM and DCE‐MRI.^13^ Though several prior studies had demonstrated comparable diagnostic performances and size measurement accuracies between CESM and DCE‐MRI, the number of participants in a single arm was inadequate. In our study, the kappa value of the BI‐RADS scores between CESM and DCE‐MRI was 0.607, indicating high correspondence between the two imaging modalities. As for lesion size measurement, CESM and DCE‐MRI had a moderate correlation with a Kendall's tau coefficient of 0.556. Our results provided additional evidence for the comparability between CESM and DCE‐MRI.

Notably, BI‐RADS 4C was used in our study as the threshold value for CESM to discriminate between benign and malignant breast lesions. However, this was not completely in line with certain previous researches in which BI‐RADS score of 4 was mostly used as the cutoff value for benign and malignant lesions.^9^, ^14^ Chaiwerawattana et al conducted a retrospective study of BI‐RADS 4 patients diagnosed in the National Cancer Institute Thailand during 2003‐2008, which showed that the malignant positive rates of 4A, 4B, and 4C patients were 7.7%, 38.7%, and 58.0%, respectively.^15^ In another study published by Elezaby et al, with 41 841 patients classified as BI‐RADS 4, the positive predictive values of 4A, 4B, and 4C patients were 7.6%, 22.0%, and 69.3%, respectively.^16^ From these results, we could infer that the malignant predictive values varied significantly among BI‐RADS four subtypes, and only BI‐RADS 4C permitted prediction of more than 50% malignant tumors. This gave us a clue to choose BI‐RADS 4C as the cutoff value for benign and malignant lesions in our study and this cutoff value exhibited high specificity and negative predictive value, indicating CESM could be recommended as a potential exclusion diagnosis modality. As mentioned before, CESM of our study was carried out only when definite diagnoses couldn't be made by other examinations. Of the 36 dense breast patients with no pathological results, 24 patients (24/30, 80.0%) were scored less than 4C, assumed to be probably benign, and were recommended for routine follow‐up. No malignant lesion was detected during the follow‐up periods, which revealed the exclusion potential of CESM indirectly.

In our study, 36 patients (36/114, 31.6%) who had pathologically confirmed adenosis with or without fibroadenoma showed no visible mass, focal asymmetry, architectural distortion, calcification, or abnormal enhancement on CESM. Adenosis is a benign glandular proliferative disease composed of epithelium, myoepithelium, and connective tissue originated from the terminal ductal lobular unit. It is widely believed that excessive estrogen over progesterone contributes to the development of adenosis. Adenosis is a process of mammary dysplasia which consisted of various histopathological subtypes. However, each subtype may exhibit overlapping imaging findings making it hard to make a correct diagnosis.^17^, ^18^, ^19^ Chen et al retrospectively reviewed 136 patients who underwent mammography in their center and were confirmed as sclerosing adenosis, a subtype of adenosis. Ten of 136 (7.4%) patients got negative results.^17^ Fewer CESM researches focused on adenosis diagnosis, but experience from contrast‐enhanced ultrasound might give us a hint. Liu et al analyzed 151 adenosis lesions and 12/151 (7.9%) lesions showed low enhancement with significant fibrous tissue proliferation and reduction in vascularity.^20^ These might partially explain the false negative cases in our study. As for the higher missed diagnosis rate (31.6%), it was probably related to the limit of our sample size and patients’ selection.

Ductal carcinoma in situ (DCIS) is a noninvasive breast neoplasm comprised of abnormal cells confined to the basement membrane. Patients with DCIS will have higher risk of developing invasive BC. Though typical cases manifest as fine‐linear branching microcalcification on mammography, data revealed by Aminololama‐Shakeri et al showed that about 10% DCIS presented as masses exclusively.^21^ Meanwhile, Cheung et al carried out a CESM research focusing on microcalcification and indicated that 2 of 15 DCIS showed no enhancement while the remaining 13 DCIS demonstrated enhancement.^22^ There were nine DCIS in our study, among which three manifested architectural distortion with calcification were classified as BI‐RADS 4C, while the rest presented as mass with enhancement of different degrees were classified as BI‐RADS 4A (1 patient), 4B (2 patient), 4C (2 patient) or 5 (1 patient). These revealed a heterogenous imaging profile of DCIS on mammography and CESM which might in part be attributable to its histopathologic type and grade. This further highlighted the value of combining different imaging modalities in achieving higher diagnostic accuracies.

Our study had several limitations. First, the sample size was small. Though our results were of statistical significance, the small number might have limited its clinical implication. Second, the single‐center nature of this retrospective study may have introduced substantial selection bias. Third, as a recently introduced technique, the diagnostic experience of CESM was insufficient and all image interpretations were based on qualitative analysis. Therefore, future multicenter large‐scale studies are warranted to validate our current promising results and to further explore the diagnostic advantages of CESM, particularly in a quantitative manner.

## 5 CONCLUSION

CESM demonstrated excellent overall diagnostic accuracy and a moderate correlation in lesion size estimation against DCE‐MRI in dense breast patients, supporting it to be an alternative to DCE‐MRI in breast cancer detection and diagnosis, especially for exclusion diagnosis.

## CONFLICT OF INTEREST

The authors report that they have no conflict of interest.

## AUTHOR CONTRIBUTIONS

Yun Qin: Paper writing and data analysis; Ying Liu: Clinical and pathological data collection and integration; Xueqin Zhang: CESM photography; Shuang Zhao, Huanhuan Zhong and Juan Huang: Imaging evaluation; Jianqun Yu: Supervision and paper revision.

## Data Availability

The data that support the findings of this study are available from the corresponding author upon reasonable request.
